# *C. elegans* CEP-1/p53 and BEC-1 Are Involved in DNA Repair

**DOI:** 10.1371/journal.pone.0088828

**Published:** 2014-02-20

**Authors:** Sandy Hoffman, Daniel Martin, Alicia Meléndez, Jill Bargonetti

**Affiliations:** 1 Department of Biological Sciences, Hunter College, City University of New York, New York City, New York, United States of America; 2 Department of Biological Sciences Queens College, City University of New York, Queens, New York, United States of America; 3 The Graduate Center Departments of Biology and Biochemistry, City University of New York, New York City, New York, United States of America; University of Hawaii Cancer Center, United States of America

## Abstract

p53 is a transcription factor that regulates the response to cellular stress. Mammalian p53 functions as a tumor suppressor. The *C. elegans* p53, *cep-1*, regulates DNA-damage induced germline cell death by activating the transcription of *egl-1* and *ced-13*. We used the *C. elegans* model to investigate how, in the whole animal, different forms of DNA damage can induce p53-dependent versus p53-independent cell death and DNA repair. DNA damage was induced by ultraviolet type C (UVC) radiation, or 10-decarbamoyl mitomycin C (DMC, an agent known to induce mammalian p53-independent cell death). Wild-type or *cep-1* loss-of-function mutant animals were assayed for germline cell death and DNA lesions. Wild-type animals displayed greater removal of UVC-lesions over time, whereas *cep-1* mutant animals displayed increased UVC-lesion retention. The *cep-1* mutation increased UVC-lesion retention directly correlated with a reduction of progeny viability. Consistent with DMC inducing p53-independent cell death in mammalian cells DMC induced a *C. elegans* p53-independent germline cell death pathway. To examine the influence of wild-type CEP-1 and DNA damage on *C. elegans* tumors we used *glp-1(ar202gf)/Notch* germline tumor mutants. UVC treatment of *glp-1* mutant animals activated the CEP-1 target gene *egl-1* and reduced tumor size. In *cep-1(gk138);glp-1(ar202gf)* animals, UVC treatment resulted in increased susceptibility to lesions and larger tumorous germlines. Interestingly, the partial knockdown of *bec-1* in adults resulted in a CEP-1-dependent increase in germline cell death and an increase in DNA damage. These results strongly support cross-talk between BEC-1 and CEP-1 to protect the *C. elegans* genome.

## Introduction

The tumor suppressor protein p53 is a transcription factor involved in activating cell cycle arrest, apoptosis, autophagy, and DNA repair [1]. The *Caenorhabditis elegans* p53-1, CEP-1, is an ancient ortholog of p53 with a conserved DNA binding domain that includes the residues often mutated in human cancers [2], [3]. In human cells, wild-type p53 is maintained at low levels, however, following DNA damage, p53 is stabilized and the increased levels result in the activation of down-stream target genes [4]. In *C. elegans*, CEP-1 is activated by DNA damage, initiated by ultraviolet C (UVC) light, ionizing radiation (IR) and N-ethyl-N-nitrosourea (ENU) [5]. CEP-1 induces germline cell death by activating the transcription of the target genes *egl-1* and *ced-13* [5], [6]. EGL-1 and CED-13 are orthologs to the human BH3 domain proteins Puma and Noxa [7], [8]. Activation of *egl-1* and *ced-13* results in germline, but not somatic, cell death [3]. UVC induced cell death in *C. elegans* also requires the nucleotide excision repair (NER) pathway [9]. The involvement of CEP-1 has been examined for apoptotic cell death but if CEP-1 or how CEP-1 participates in autophagic cell death has not been examined.

Autophagy is a self-eating signaling cascade that is used for both cell survival and cell death [10]. A critical regulator of autophagy in mammals is the protein Beclin 1 [11], [12]. BEC-1 is the *C. elegans* ortholog of mammalian Beclin 1, and is required for viability, fertility, growth, dauer development and survival [12], [13], [14]. BEC-1 interacts directly with, and regulates, CED-9/Bcl-2 [14]. There is a complex connection between BEC-1 and the induction of cell death. High levels of TUNEL staining, indicative of an accumulation of DNA damage, occur in *bec-1* null animals [14]. Furthermore, an increase in germline cell death has been noted in *bec-1* null mutants and *bec-1* RNAi fed animals, and this increase occurs at least in part due to a delay in apoptotic cell corpse degradation [14], [15].

The *C. elegans* germline cell death is increasingly used as a model system to study human cancer associated signaling events [16], [17], [18]. The cross-talk between CEP-1, BEC-1, and DNA repair is an under-studied area. Investigating the highly proliferative germ cells in *C. elegans*, and resistance to DNA damage in germline-tumor phenotype animals, can help us understand how mammalian tumors respond to DNA damage signaling. Localized GLP-1/Notch signaling controls the mitotic proliferation that occurs in the distal part of the gonad, even during adulthood [19]. The GLP-1/Notch receptor is activated by the LAG-2 ligand, which is produced by the Distal Tip Cell (DTC) to keep cells in the distal region of the gonad in mitosis. As germ cells move away from the DTC signal, they lose the signal and transition into meiosis. Thus, the GLP-1/Notch receptor controls mitosis and allows for the inhibition of meiosis or differentiation. GLP-1/Notch constitutive gain-of-function mutants result in a germline-tumor phenotype, where germ cells persist in mitosis and do not enter meiosis [19], [20]. This gain-of-function causes hyper-proliferation of the germ line which presented us with a source of actively proliferating cells in a multicellular model. We have used the *C. elegans* multicellular eukaryote as a model to evaluate the requirement for CEP-1 on DNA repair, and signaling for cell death, in wild-type and germline-tumor animals.

UVC radiation causes dose dependent DNA lesions in wild-type *C. elegans* [21]. Using wild-type and *cep-1(gk138)* mutant animals, we compared the activation of p53 target genes and the efficiency of DNA-lesion removal, following DNA damage, initiated by UVC and the chemotherapeutic 10-decarbamoyl mitomycin C (DMC). DMC is a mitomycin analogue that initiates a robust mammalian p53-independent cell death signal [22], [23]. We measured DNA damage and repair in wild type and *cep-1(gk138)* mutant animals after UVC or DMC treatment. We hypothesized a genetic dependency for CEP-1 to repair bulky DNA-lesions, and therefore, expected that in the absence of CEP-1, we would observe an increase in DNA-lesions. Indeed, in the absence of CEP-1, we observed a reduction in the DNA repair of UVC induced lesions. Interestingly UVC exposure induced only CEP-1/p53-dependent cell death, while DMC treatment induced cell death that did not require CEP-1/p53. Moreover, we found that a partial loss of *bec-1* induced CEP-1/p53-dependent germline cell death while only slightly activating CEP-1/p53 target genes, and depletion of *bec-1* in *cep-1* mutant animals throughout development resulted in exacerbated UVC induced lesions. These data indicate that CEP-1 plays a role in removing DNA damage and that the loss of BEC-1 sensitizes worms to increased DNA damage. This suggests that CEP-1 and BEC-1 cross-talk to facilitate robust DNA repair to protect the *C. elegans* genome.

## Results

### *cep-1(gk138)* Mutants Exhibit UVC-induced Nuclear DNA Damage Lesions

**Figure 1:**
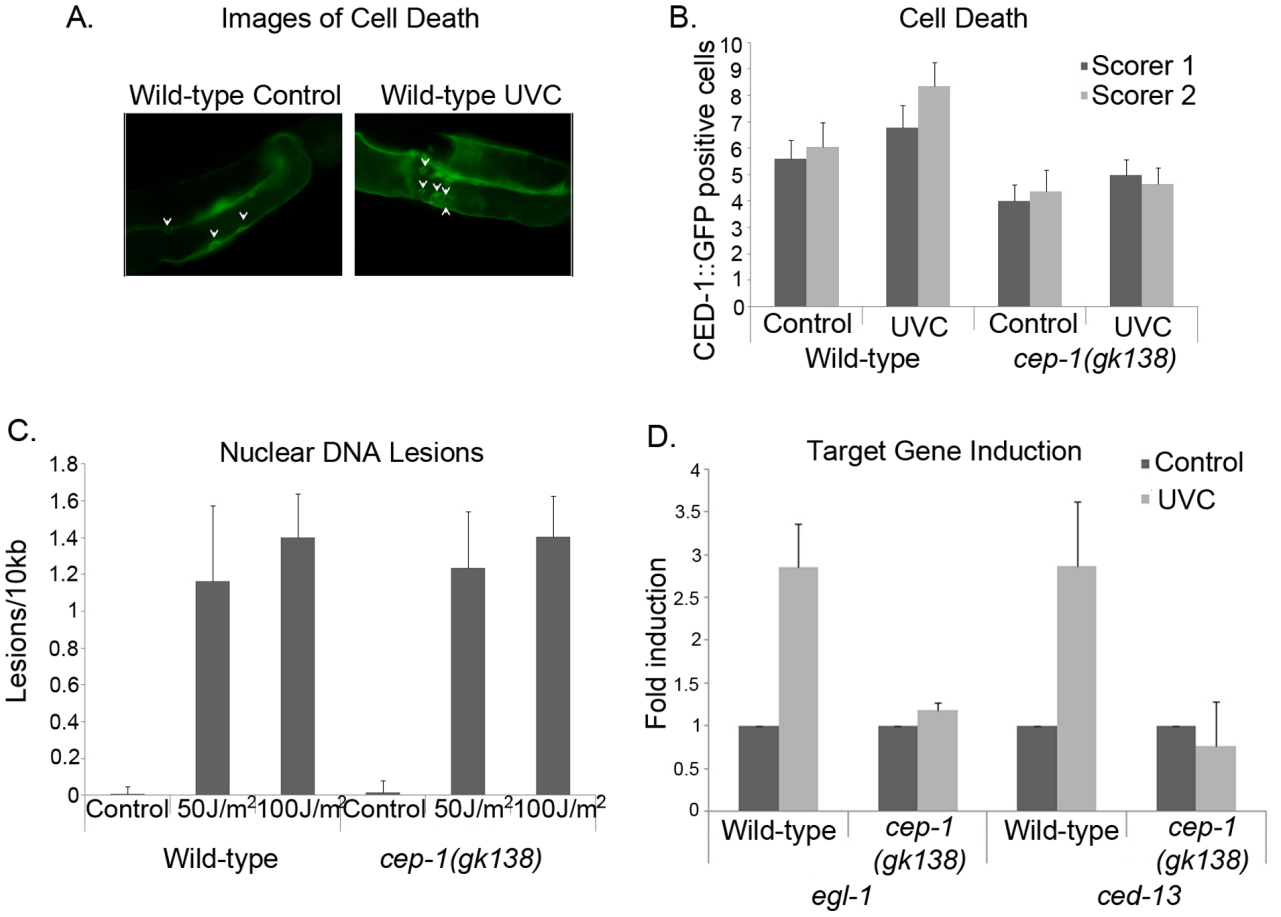
Wild-type and *cep-1(gk138)* mutant worms had similar amounts of nuclear DNA damage after UVC exposure. **A)** Images of CED-1::GFP positive cells of wild-type worms either untreated or 24 hours after 100 J/m^2^ UVC. Arrows point to CED-1::GFP positive cells. Magnification is 400x. **B)** Worms were treated 24 hours post L4 and imaged 24 hours later. The number of CED-1::GFP positive cells in the germ line was scored blindly by two independent people. Error bars indicate standard error and the number of worms scored was 20. The difference between wild-type untreated and UVC treated animals had a P value were all equal to or less than 0.002. **C)** Analysis of nuclear DNA lesions on 24 hour post L4 wild-type or *cep-1(gk138)* mutant animals, after exposure to 50 or 100 J/m^2^ of UVC. Average of three representative experiments is shown. Error bars indicate standard error. The wild-type P values were 0.0001 and 0.003 for a comparison to untreated at 50 or 100 J/m^2^ respectively. The *cep-1(gk138)* mutant P values were 0.012 and 0.02 for a comparison to untreated at 50 or 100 J/m^2^ respectively. **D)** Q-RTPCR of *egl-1* and *ced-13* mRNA levels four hours post UVC treatment of 100 J/m^2^. Fold induction compared to cDNAs amplified from untreated worms. Average of three representative experiments is shown. Error bars indicate standard error. Normalized to *tbg-1*. The P values for wild-type animals were less than or equal to 0.02 for both *egl-1* and *ced-13.* No significant change was detected in *cep-1(gk138)* mutant animals.

While the influence of UVC radiation on DNA lesions and cell death has been examined in wild-type animals, the influence on *cep-1(gk138)* mutant animals is an under-studied area [24]. It is well established that UVC damage induces germ cell death in wild-type *C. elegans*, activates CEP-1 to initiate the transcription of *egl-1* and *ced-13,* and uses the nucleotide excision repair pathway in this process [7], [8], [24]. In keeping with the published literature, only wild-type animals displayed a significant UVC-induced increase in germline cell death (Fig. 1B) [24]. The previously published work used Nomarski optics to score cell corpses per gonad arm. We examined how the loss of CEP-1 influenced UVC induced DNA damage and cell death by exposing young adult animals to UVC radiation and using a CED-1::GFP reporter to score for germline cell death (Fig. 1A). To eliminate any possible bias, we had two different individuals blindly score the number of CED1::GFP engulfed cell corpses. Using CED1::GFP positive cells we observed a significant increase in cell death with a P value of 0.002; however the values of corpses per gonad arm were lower than those previously published that were obtained with Nomarski optics [24]. To examine the influence of CEP-1 on UVC-induced DNA damage, we used the PCR-based lesion detection method (introduced and validated by the Meyer laboratory [21]). This lesion assay measures DNA damage via the inhibition of PCR amplification of specific mitochondrial and nuclear genes. UVC treatment causes a drastic increase in the lesions detected per 10 kb, when compared to untreated animals [21], [25]. We predicted that UVC treated wild-type, and *cep-1(gk138)* mutant animals would sustain similar levels of UVC-induced DNA lesions. As expected, both wild-type and *cep-1(gk138)* young adult animals displayed a robust increase in nuclear DNA lesions (Fig. 1C).

DNA damage lesions are the initial stimulus that activates the p53-pathway. We confirmed that UVC-induced DNA lesions activated CEP-1 target genes in wild-type animals, with the *egl-1* and *ced-13* induction levels increased in young adults, four hours post UVC treatment. As previously reported [24], no increase in either *egl-1* or *ced-13* was observed following UVC treatment of the *cep-1(gk138)* mutants (Fig. 1D).

### CEP-1 Allows for the Removal of UVC-induced DNA-lesions thus Facilitating DNA Repair

**Figure 2:**
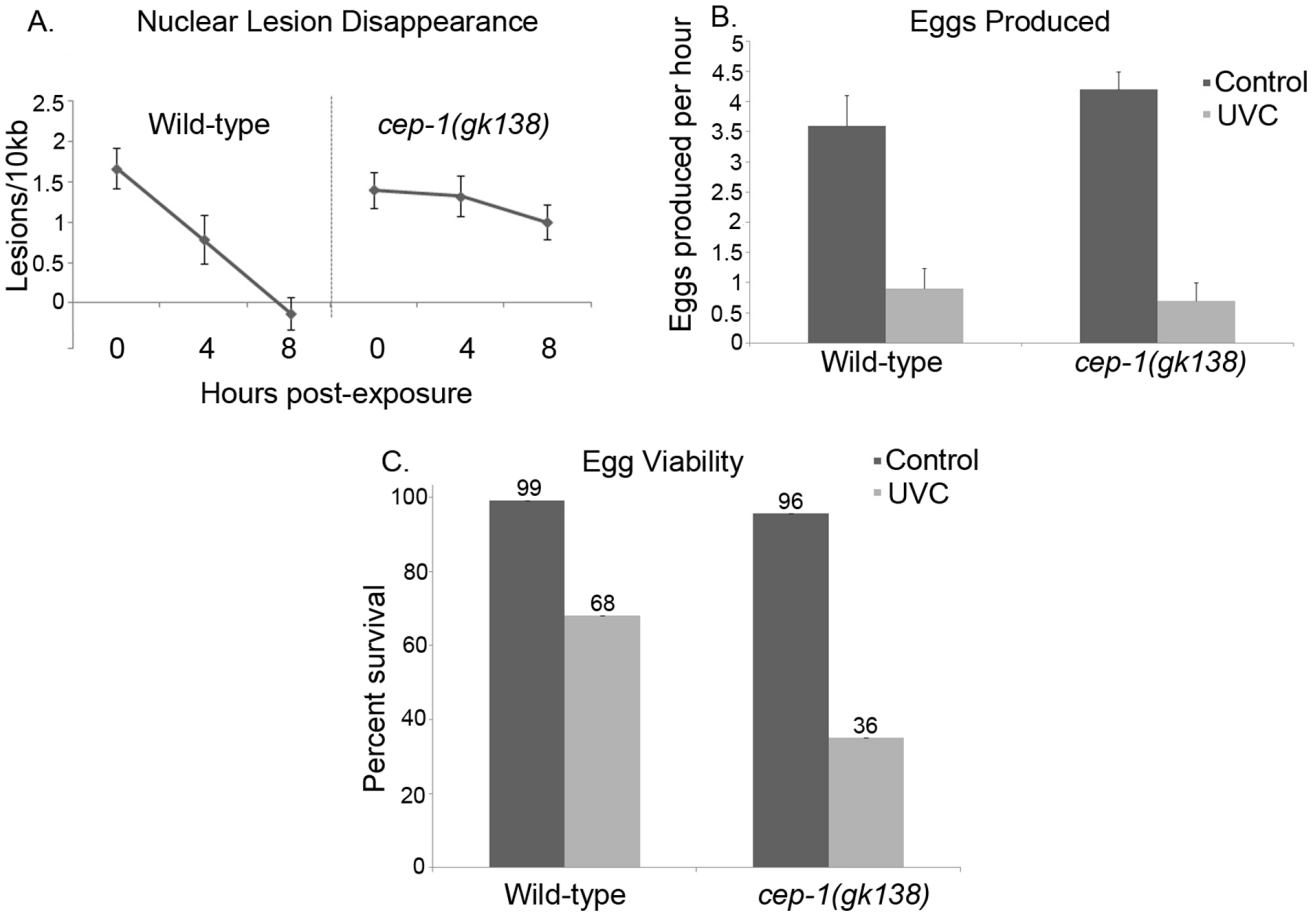
*cep-1(gk138)* mutants had less repair and decreased egg viability than wild-type worms after UVC treatment. **A)** Analysis of nuclear DNA lesions at 0, 4 h or 8 h time points of post L4 wild-type or *cep-1(gk138)* mutant animals after exposure to 100 J/m^2^ of UVC. Average of three representative experiments is shown. Error bars indicate standard error. Significant lesions were observed in wild-type worms with a P value of 0.0002 at the zero hours and 0.03 after four hours of repair when compared to untreated wild-type worms. The P values for *cep-1(gk138)* mutant animals were equal or less than 0.03 for each time point when compared to untreated animals. **B)** Analysis of the rate of egg laying of animals treated with UVC as L4 and allowed to recover for 24 hours. Egg laying values represent number of eggs laid per hour per adult hermaphrodite. Average of 18 worms are shown. Error bars indicate standard error. P values were less than or equal to 0.003 for both strains when compared to untreated animals. **C)** Percent survival of eggs from wild-type or *cep-1(gk138)* mutant animals after no treatment or exposure to UVC as L4s and allowed to recover for 24 hours. Survival of eggs was determined as the number of eggs that hatched after 1 day over the total number of eggs laid after 24 h. P values were less than or equal to 0.001 for both strains when compared to untreated animals.

Mammalian p53 is involved in DNA repair by activating downstream targets that assist in the nucleotide excision repair (NER) pathway [26], [27], [28], [29]. p53 deficient mouse embryonic fibroblast cells(MEFs) have less DNA repair when compared to cells with wild-type p53 after UVC treatment [28], [30]. However, the role of CEP-1 in DNA repair has yet to be well characterized. UVC DNA damage induces DNA lesions in *C. elegans* that are measurable by PCR analysis, and, in wild-type animals, these lesions are rapidly repaired [21]. We examined the influence of CEP-1 on *C. elegans* DNA repair, by measuring nuclear DNA lesions four and eight hours after UVC treatment. We observed repair in wild-type worms, since after eight hours very few lesions remained (Fig. 2A). The *cep-1(gk138)* mutants, on the other hand, demonstrated very minor removal of the UVC-induced DNA lesions (Fig. 2A). This indicated that CEP-1 was required for efficient nuclear DNA repair.

*C. elegans* with defects in DNA damage repair display a reduction in the number of viable eggs laid after DNA damage [24], [31], [32]. A decrease in egg laying, or egg survival, can indicate DNA damage that is not repaired. To further confirm that CEP-1 played a role in DNA repair, we scored the number of eggs laid and percent egg survival in UVC exposed wild-type and *cep-1(gk138)* mutant animals. Both, wild-type and *cep-1(gk138)* mutant animals, treated with UVC, produced less eggs (Fig. 2B), and the viability of eggs laid by both strains decreased after UVC treatment (Fig. 2C). Importantly, while wild-type worms had 68% egg survival following DNA damage, *cep-1(gk138)* mutants had only 36% egg survival (Fig. 2C). Thus, low egg survival for CEP-1 mutant animals correlated with a decreased capacity to repair UVC-induced lesions.

### DMC Induces DNA Damage and CEP-1-independent Cell Death in *cep-1(gk138)* Mutant Animals

**Figure 3:**
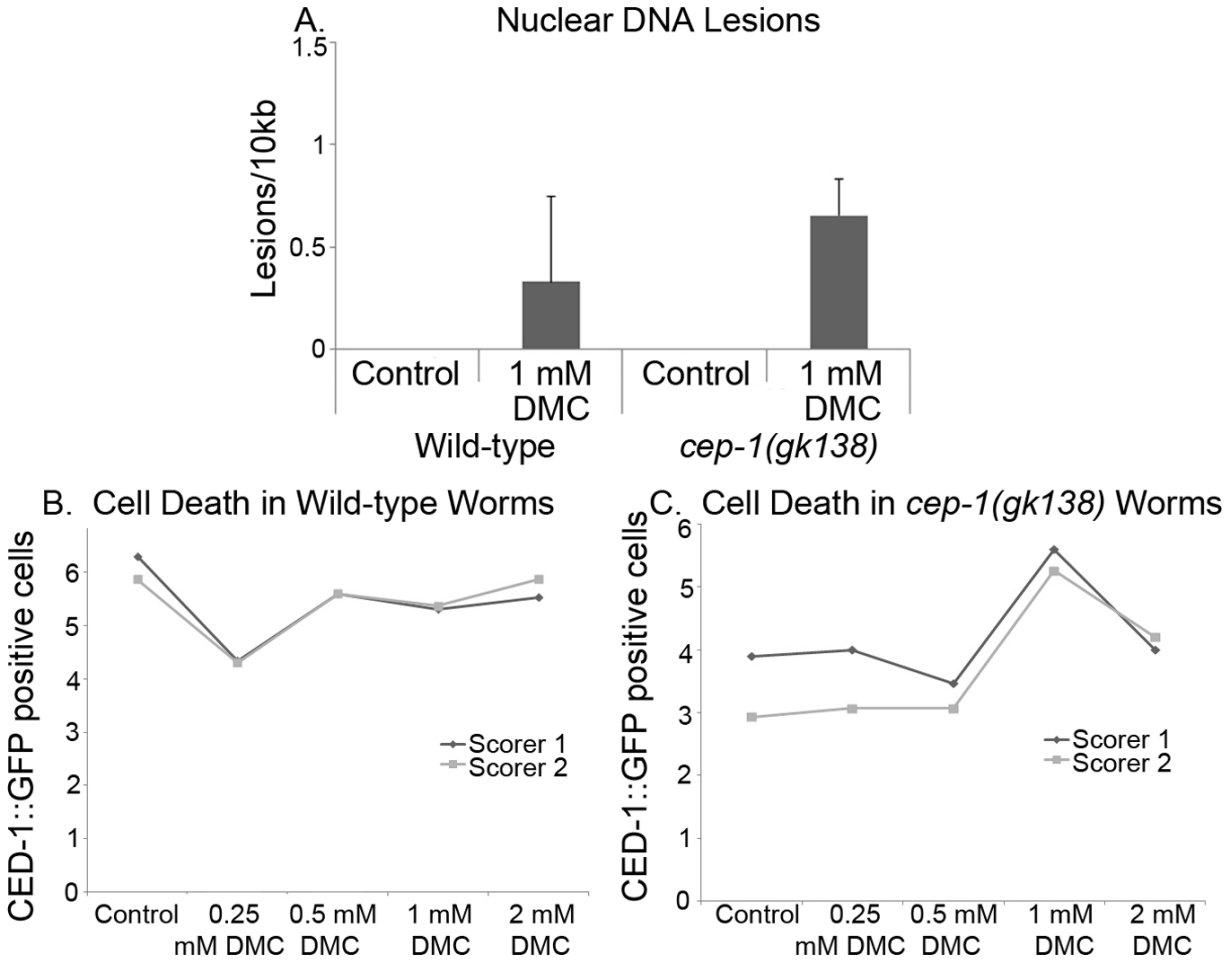
DMC caused an increase in lesions and increased germline cell death in *cep-1(gk138)* mutant worms. **A)** Lesions of nuclear DNA were measured on 24 hour old adult wild-type or *cep-1(gk138)* mutant animals, after five hours of treatment with 1 mM DMC or 30% methanol for 5 hours (as L4 larvae). The lesions detected were not statistically significant. **B)** Average of three representative experiments is shown. Error bars indicate standard error. The cell death compared to the control was not statistically significant at 0.5, 1 and 2 mM. The number of dead CED-1::GFP positive cells in the germ line was determined in wild-type adults or in **C)** *cep-1(gk138)* mutant animals after feeding with 30% methanol or increasing concentrations of DMC (0.25 mM, 0.5 mM, 1.0 mM or 2.0 mM DMC), overnight. Only 1 mM DMC treatment demonstrated statistically significant cell death with a P value of 0.02. The number of CED-1::GFP positive cells was scored blindly by two independent people. The number of worms scored per point was 20.

DMC is an alkylating agent that results in a high frequency of bulky DNA-adducts in human cells and causes p53-dependent and p53-independent cell death [22], [23]. We asked if, in *C. elegans,* DMC could induce nuclear DNA damage and germline cell death. 1 mM DMC was placed on plates and fed to animals for five hours, and nuclear DNA lesions were measured. While DMC is very toxic to breast cancer cells grown in culture [22], [23], *C. elegans* were rather resistant to the drug. *C. elegans* are impermeable to many chemotherapeutic drugs due to the animals thick cuticle and this has limited the model from being used for many drug studies [33]. Treatment with DMC caused barely detectable DNA damage in wild-type animals and only moderate lesion detection in *cep-1(gk138)* mutants (Fig. 3A). DMC treatment caused less than 0.5 lesions per 10 kb, and in *cep-1(gk138)* mutants this was only increased to slightly above 0.5 lesions per 10 kb. In both cases the lesions per 10 kb dropped lower when followed over time (data not shown). After an overnight treatment with varying DMC concentrations, CED-1::GFP positive cells were scored in wild-type and *cep-1(gk138)* animals to determine if there was an increase in cell death. Wild-type animals did not show an increase in CED-1::GFP positive cells, but with 1 mM DMC treatment the *cep-1(gk138)* mutants displayed a reproducible increase in cell death (Fig. 3C). This cell death was reduced at 2 mM DMC, potentially due to activation of xenobiotic efflux pumps [33]. DMC treatment did not affect the number of eggs laid by either of the two strains. The *cep-1(gk138)* mutants displayed a decrease in egg survival after DMC treatment (data not shown). Taken together these data suggest that the very few lesions induced by DMC resulted in more of a synthetic lethality when there is no CEP-1 expressed in the *cep-1(gk138)* mutant animals. The molecular pathway for DMC induced p53-independent cell death remains under investigation.

### CEP-1 can be Activated in *glp-1(ar202gf)* Tumor Mutant Animals and *cep-1(gk138); glp-1(ar202gf)* Tumor Mutant Worms have Increased Nuclear Lesions after DNA Damage

**Figure 4:**
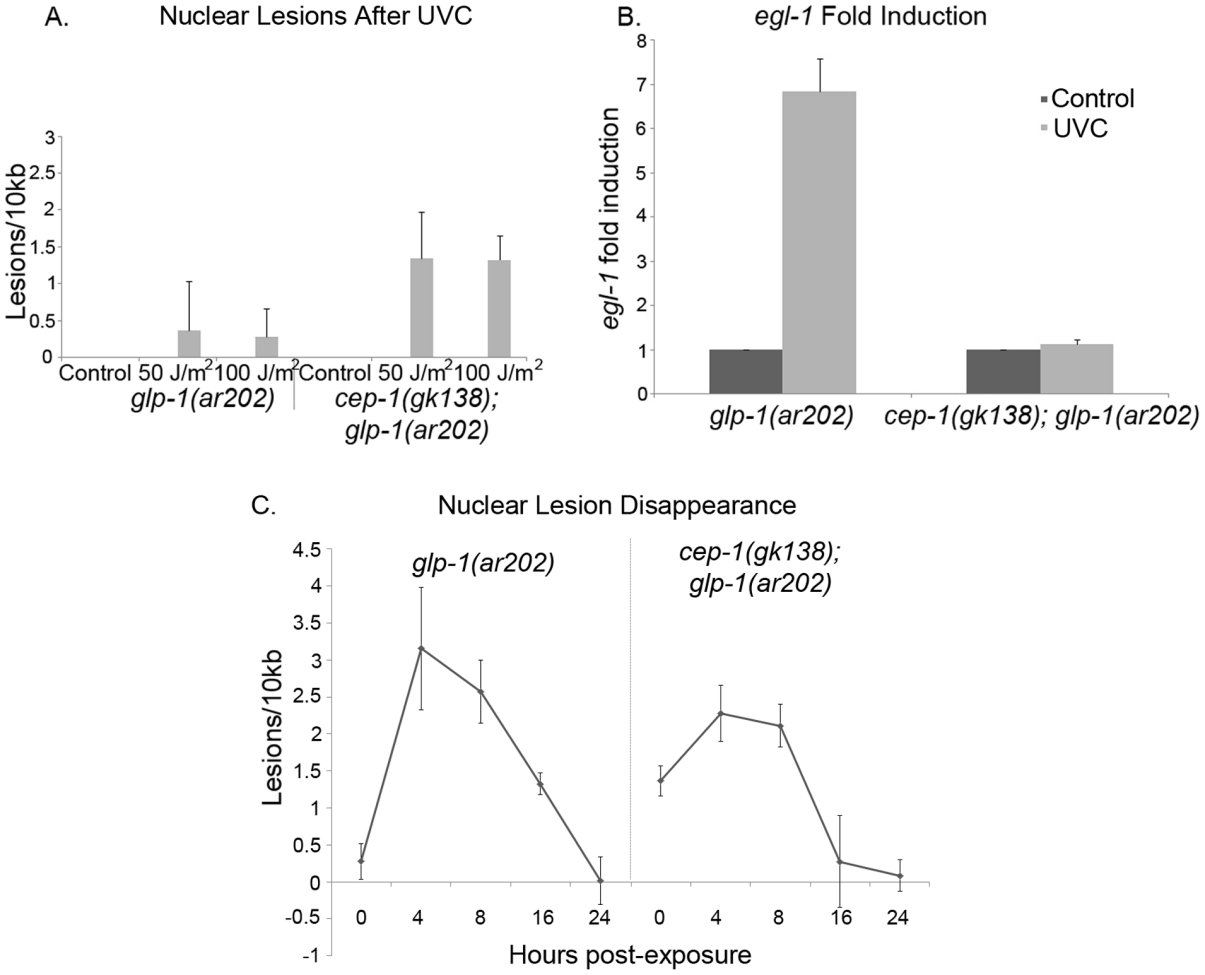
CEP-1 was activated in *glp-1(ar202gf)* tumor mutant worms. **A)** Nuclear DNA lesions were measured in *glp-1(ar202gf)* or *cep-1(gk138); glp-1(ar202gf)* double mutants, immediately following UVC exposure. Animals were exposed to 50 or 100 J/m^2^ of UVC, as adults (24 h post L4 stage). Average of three representative experiments is shown. Error bars indicate standard error. Only *cep-1(gk138); glp-1(ar202gf)* double mutants had a statistically significant increase in lesions with P value of 0.003 after 100 J/m^2^. **B)** Expression of *egl-1* mRNA determined by Q-RTPCR in *glp-1(ar202gf)* or *cep-1(gk138); glp-1(ar202gf)* double mutant animals, 4 hours post UVC treatment of 100 J/m^2^. Data are reported as fold induction compared to expression in untreated worms and normalized to *tbg-1*. Average of six representative experiments is shown. Error bars indicate standard error. The *egl-1* fold change had a P value in *glp-1(ar202gf)* of 0.008 and the P value in *cep-1(gk138); glp-1(ar202gf)* double mutants was 0.03. **C)** Nuclear lesions were quantified immediately, four, eight, sixteen and twenty-four hours after UV exposure in *glp-1(ar202gf)* and *cep-1(gk138); glp-1(ar202gf)* worms. Average of three representative experiments is shown. Error bars indicate standard error. In *glp-1(ar2020gf)* mutant worms significant lesions were detected with the P values equal or less than 0.02 for time points four, eight and sixteen hours after UVC treatment when compared to untreated animals. In *cep-1(gk138); glp-1(ar202gf)* mutant worms significant lesions were detected with the P values equal or less than 0.004 for time points zero, four and eight hours after UVC treatment when compared to untreated animals.

**Figure 5:**
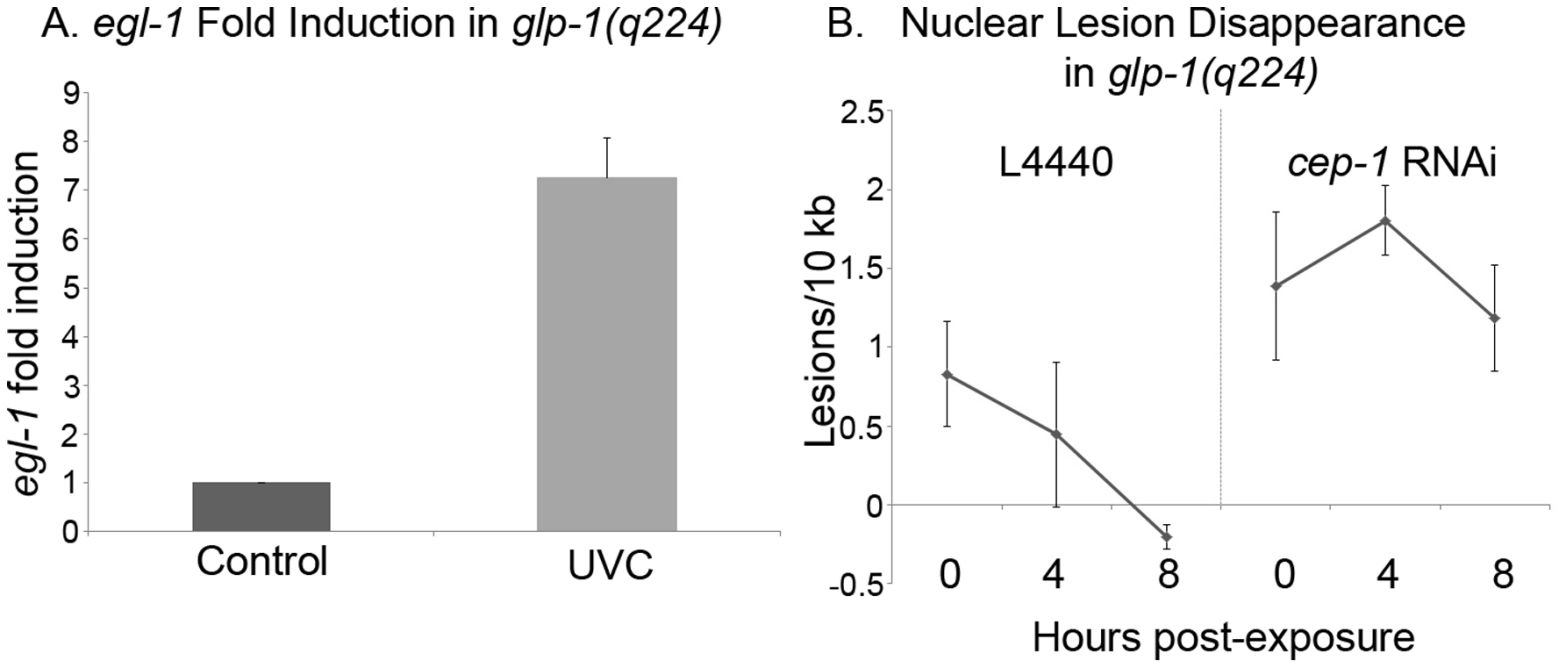
CEP-1 acts to remove UVC induced lesions in somatic cells. **A)** *egl-1* expression measure by Q-RTPCR in *glp-1(q224lf)* animals that lack a germline, 4 hours post UVC treatment of 100 J/m^2,^ or control animals without treatment. Data are reported as fold induction compared to expression in untreated worms and normalized to *tbg-1*. Average of two representative experiments is shown. Error bars indicate standard error, P value of 0.008. **B)** Nuclear DNA lesions were measured immediately, four and eight hours after 100 J/m^2^ of UVC in *glp-1(q224lf)* worms grown on either L4440 or *cep-1* RNAi plates at 25°C. Average of three representative experiments is shown. Error bars indicate standard error. Wild-type animals at 0 hours had significant lesions with a P value of 0.03 and no significant lesions at 4 and 8 hours. *cep-1* depleted animals had significant lesions at 0, 4, and 8 hours after UVC treatment with P values of equal to or less than 0.05.

We were interested in determining if CEP-1 played a role in the apparent resistance to DNA damage of *glp-1/Notch* germline-tumor mutants (maintained at 25°C). To address this, we examined the gain of function *glp-1(ar202gf)* mutant animals and constructed *cep-1(gk138); glp-1(ar202gf)* double mutant animals. Both genotypes produced almost 100% offspring with a Tumorous (Tum) germline at 25°C. We measured nuclear DNA damage in *glp-1(ar202gf)* and *cep-1(gk138); glp-1(ar202gf)* double mutant animals that displayed the Tum phenotype. Immediately after 50 and 100 J/m^2^ of UVC treatment, the *glp-1(ar202gf)* mutant animals had fewer nuclear DNA lesions than the *cep-1(gk138); glp-1(ar202gf)* animals (Fig. 4A). To determine if this increase in DNA lesions correlated with the expected decrease in CEP-1 activity we scored for *egl-1* fold induction. We treated *glp-1(ar202gf)* and *cep-1(gk138); glp-1(ar202gf)* animals with 100 J/m^2^ of UVC and measured *egl-1* fold induction four hours later. UVC treatment of *glp-1(ar202gf)* induced *egl-1* expression, while the same treatment of *cep-1(gk138); glp-1(ar202gf)* double mutant animals did not (Fig. 4B). Therefore, just as in wild-type worms, *glp-1(ar202gf)* worms contained CEP-1 protein that was transcriptionally active and that participated in DNA repair. Clearance of the DNA lesions for the treated *glp-1(ar202gf)* and *cep-1(gk138); glp-1(ar202gf)* animals showed an intriguing result that supports a previous report of a UVC-mediated coordination between the nucleotide excision repair pathway (NER) and the homologous repair pathway (HR) [34]. When we examined lesion clearance in UVC treated *glp-1(ar202gf)* mutants and *cep-1(gk138); glp-1(ar202gf)* double mutants we observed an increase in lesions four hours after treatment with a subsequent decrease by 24 hours in both cases. In *cep-1(gk138); glp-1(ar202gf)* double mutants we documented that UVC treatment caused a substantial increase in lesions to above 1.5 per 10 kb (Fig. 4A); we observed an increase in lesions per 10 kb at four hours after UVC treatment and all the lesions were absent after 24 hours (Fig. 4C). We reasoned that the high rate of DNA replication in the *cep-1(gk138); glp-1(ar202gf)* double mutant mitotic germline allowed for increased homologous recombination repair in these animals. This is supported by Figure 5, which demonstrated that the absence of a germline yields animals that show efficient UVC lesion clearance in the presence of CEP-1 but not its absence (see below).

### CEP-1 has a Role in DNA Damage Repair in Germlineless Animals

The primary published role for CEP-1 is the induction of germ cell death after DNA damage [3]. When CEP-1::GFP is exogenously expressed, CEP-1 is only localized to the pharynx and the germ line of adult animals [3]. While this suggests that CEP-1 may not play a role in the somatic cells of adult worms, one-third of the total number of cells in the adult hermaphrodite, are somatic cells. To investigate the UVC DNA damage response in somatic cells, we investigated animals that lacked a germline. We used the *glp-1(q224lf)* mutant animals that are deficient in the germ line and exposed them to 100 J/m^2^ of UVC and measured for *egl-1* expression. We observed that the *glp-1(q224lf)* mutant animals displayed *egl-1* induction (Fig. 5A), suggesting that *egl-1* expression, after UVC induced DNA damage, also occurred in somatic cells. We next asked if CEP-1 played a role in the removal of UVC induced lesions in the *glp-1(q224lf)* mutant animals. Following exposure to 100 J/m^2^ of UVC, and *cep-1* depletion by RNAi, nuclear lesions were measured, either immediately, or with a clearance time of four or eight hours. Immediately after treatment, there was no difference in the amount of lesions induced by UVC in the presence or absence of CEP-1 activity (Fig. 5B). The *glp-1(q224lf)* mutant worms, expressing a functional *cep-1* gene, displayed high nuclear lesion removal eight hours after UVC treatment. The animals without *cep-1* expression retained their nuclear DNA lesions (Fig. 5B). These data suggest that CEP-1 plays a role in DNA repair in somatic cells.

**Figure 6:**
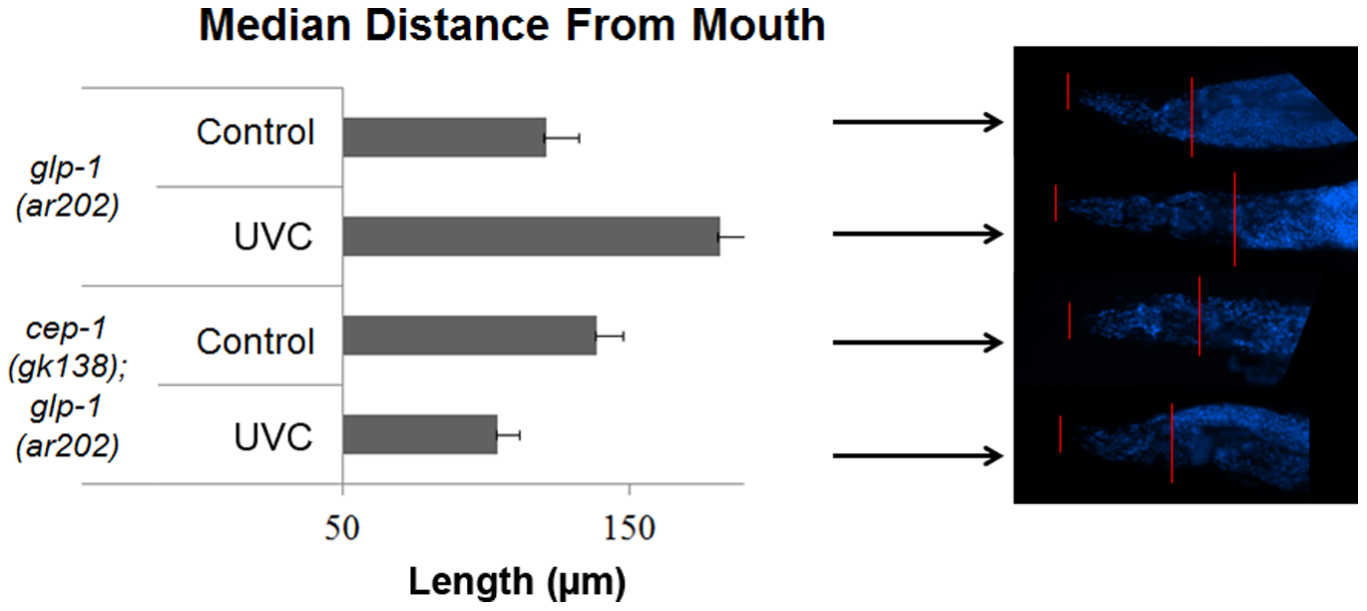
*glp-1(ar202gf)* tumor mutants displayed an increase in tumor size in the absence of *cep-1,* four days after UVC treatment. The size of the germ line tumors was determined in *glp-1(ar202gf)* or *cep-1(gk138); glp-1(ar202gf)* double mutant animals grown at the restrictive temperature after treatment with 100 J/m^2^ of UVC, and allowed to recover for four days. The size of the tumor is inversely proportional to the distance between the mouth and the tumor. Median distances of 18 worms are shown. *glp-1(ar202gf)* or *cep-1(gk138); glp-1(ar202gf)* double mutant animals had P values of 0.04 and 0.009 respectively for their change in tumor size versus untreated controls.

To address the role of CEP-1 in UVC induced DNA damage repair in germline tumor animals, we asked how the UVC induced lesions would influence the size of the germline-tumor in *glp-1(ar202gf)* mutant animals. We were unable to detect a robust increase in UVC-induced lesions in these animals. The tumor size of *glp-1(ar202gf)* mutants and *cep-1(gk138); glp-1(ar202gf)* double mutants was measured by how far the tumor had invaded the mouth [35]. The larger the tumor size observed, the smaller the distance between the tumor and the mouth. Interestingly, four days after UVC treatment the *cep-1(gk138); glp-1(ar202gf)* double mutants had larger tumors while the *glp-1(ar202gf)* tumor mutants had decreased tumor size (Fig. 6). We examined the induction of cell death by SYTO-12 staining of *glp-1(ar202gf)* mutants and *cep-1(gk138); glp-1(ar202gf)* double mutant animals four hours after UVC treatment and did not detect a significant difference of cell death in the two populations. This suggests that the variable tumor size could not be explained by increased cell death in the single mutant *glp-1(ar202gf)* animals.

### Partial Knockdown of *bec-1* did not Increase the Number of Apoptotic Corpses in the Absence of CEP-1

**Figure 7:**
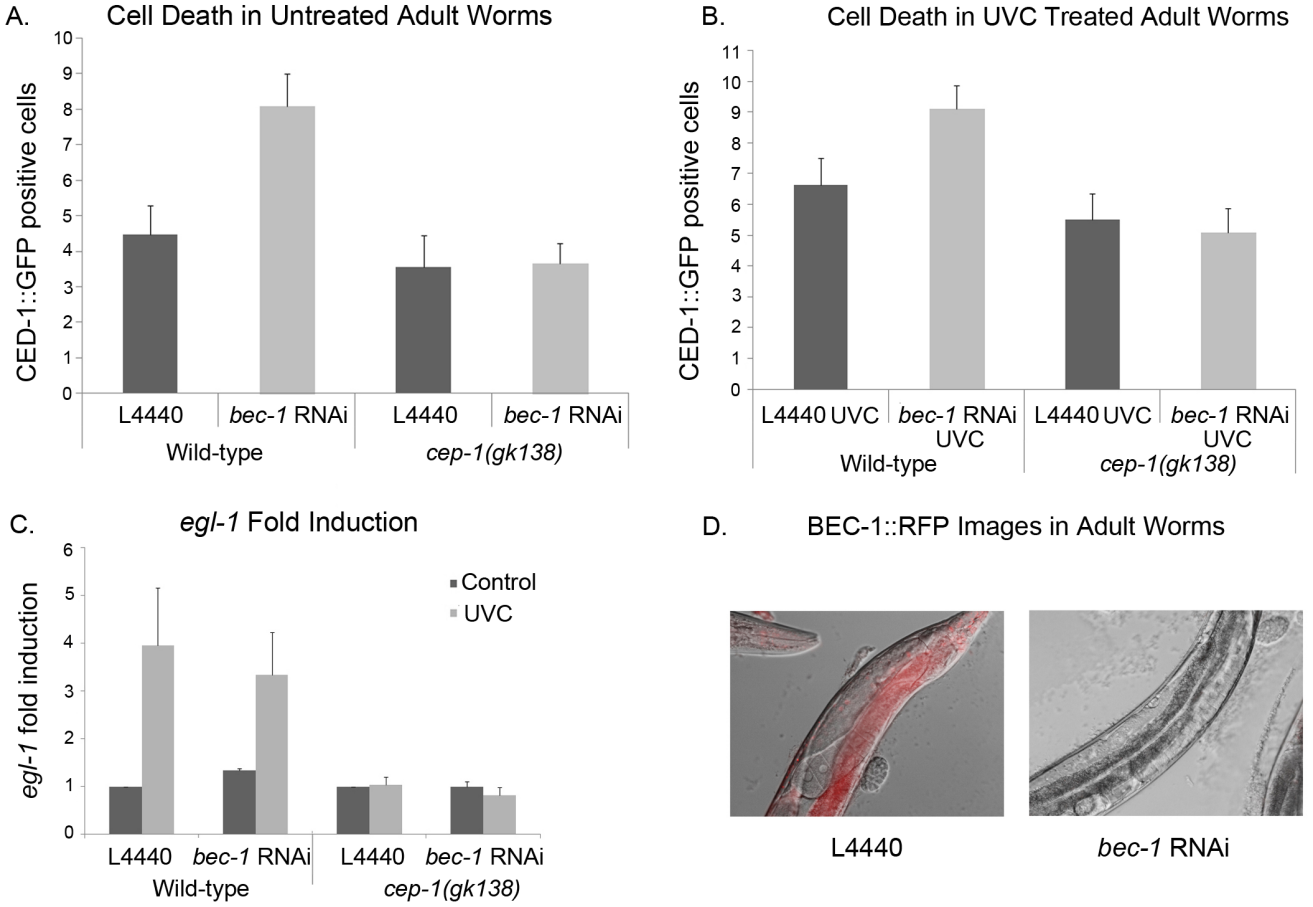
In treated adult worms, increased death from *bec-1* RNAi depletion required CEP-1. A) Animals carrying the CED-1::GFP reporter were fed control (empty vector) or *bec-1* RNAi expressing bacteria and scored for germline cell death in the absence or B) presence of 100 J/m^2^ of UVC. The number of CED-1::GFP positive dead cells was scored blindly by two independent people. Error bars indicate standard error and the number of worms scored per bar was 20. In wild-type worms, the knockdown of *bec-1* caused a significant increase of CED-1::GFP positive cells with P values of equal or less than 0.003 when compared to untreated animals. C) Expression of *egl-1* was detected by Q-RTPCR, 4 hours post 100 J/m^2^ of UVC treatment. Data are reported as fold induction compared to expression in untreated worms and normalized to *tbg-1*. The average of three representative experiments is shown. Error bars indicate standard error. L4440 UVC induction was significant with a P value of 0.03 as was the P value for animal fed *bec-1* RNAi with a P value of 0.0004 when compared to untreated L4440 animals. No significant change was detected in *cep-1(gk138)* mutant animals. **D)** Images of BEC-1::RFP worms 48 hours after L4 worms were placed on L4440 or *bec-1* RNAi plates. The Nomarski and red channels were placed on top of each other. Magnification is 400x.

The knockdown of *bec-1,* throughout development, results in an increase in embryonic and germline cell death that has been shown to be due to a defect in apoptotic cell clearance [14], [15], [36], [37]. Partial knockdown by *bec-1* RNAi treatment of L4 larvae for 24 hours, and exposure to UVC, resulted in an increase in apoptotic germ cells that were diligently degraded and thus not due to a defect in clearance (Figs. 7, A and B). The increase in apoptotic germ cells after *bec-1* partial knockdown (in wild-type L4 larvae treated with RNAi against *bec-1*), was dependent on CEP-1 because the *cep-1(gk138)* mutant animals treated with *bec-1* RNAi as L4 larvae for 24 hours had no increase in cell death (Fig. 7A). Additionally, wild-type animals had an increase in CED-1::GFP positive cells in *bec-1* RNAi fed animals after UVC treatment while *cep-1(gk138)* mutant worms did not (Fig. 7B). This suggested that the partial *bec-1* knockdown-induced germline cell death was dependent on the presence of CEP-1. The partial knockdown of *bec-1* only slightly increased the transcriptional activity of CEP-1, from 1 to 1.3 before UVC treatment (Fig. 7C). Figure 7D shows the knockdown of BEC-1::RFP by feeding L4 worms *bec-1* RNAi for 48 hours. We then knocked down *bec-1* throughout development by also feeding *bec-1* RNAi to their F1 progeny. To determine if the increased observable germline cell death was due to an actual increase in cell death or because of faulty clearance, we followed CED-1::GFP corpses for over an hour. After an hour, some of the corpses in the control (empty vector, L4440) fed worms disappeared, while the apoptotic cells in *bec-1* RNAi fed animals were not degraded. This confirmed that the observable increase of CED-1::GFP positive cells in animals RNAi treated against *bec-1* throughout development was due to a defect in apoptotic cell clearance [15]. Additionally, this detectable increase in cell death occurred independently of CEP-1.

### *bec-1* Knockdown in Treated Adults and Progeny Increases DNA Damage

**Figure 8:**
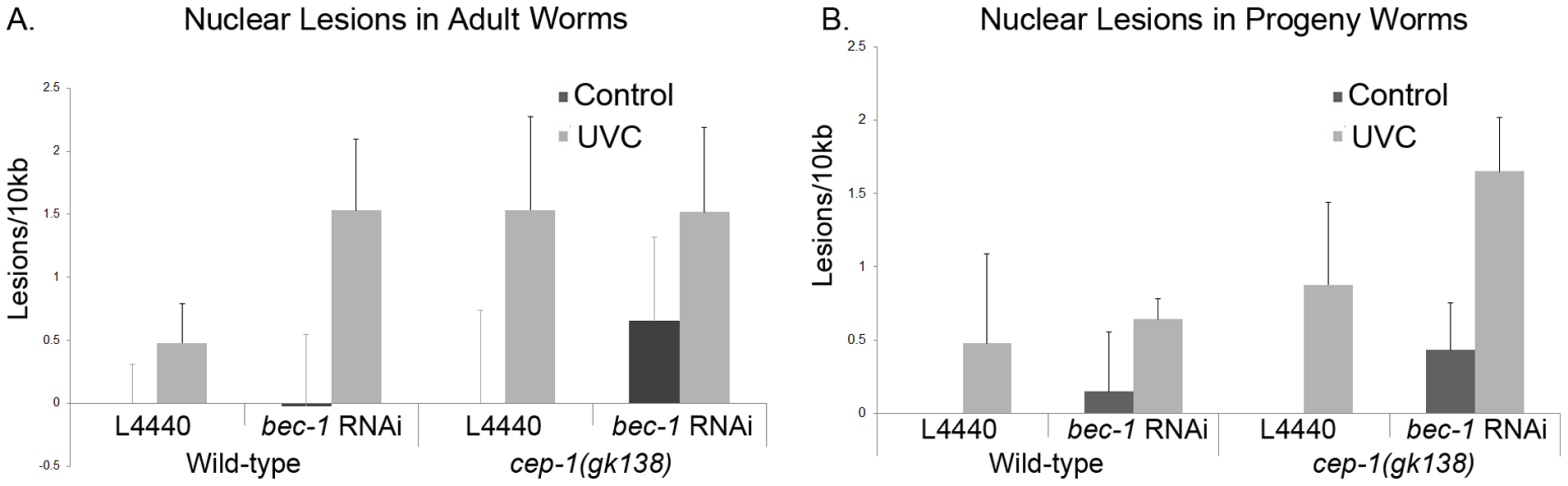
UVC treatment after *bec-1* depletion increased the number of DNA lesions in the treated adult animals. Wild-type and *cep-1(gk138)* mutant animals were exposed to 100 J/m^2^ of UVC. **A)** The number of nuclear DNA lesions were measured in adult worms fed control (empty vector) RNAi or *bec-1* RNAi expressing bacteria, during adulthood. All comparisons were made to L4440 untreated animals. For UVC treated wild-type animals fed L4440, the lesions were significantly increased with a P value of 0.04. For UVC treated wild-type animals fed *bec-1* RNAi, the lesions were significantly increased with a P value of 0.02. For UVC treated *cep-1(gk138)* mutant worms animals fed L4440, the lesions were significantly increased with a P value of 0.001. For UVC treated *cep-1(gk138)* mutant worms animals fed *bec-1* RNAi, the lesions were significantly increased with a P value of 0.004. **B)** RNAi depletion was also achieved throughout development**.** Average of three representative experiments is shown. Error bars indicate standard error. All comparisons were made to L4440 untreated animals. For UVC treated wild-type animals fed L4440, the lesions were significantly increased with a P value of 0.05. For *bec-1* knockdown without UV treatment, there was a significant increase with a P value of 0.01. For UVC treated wild-type animals fed *bec-1* RNAi, the lesions were significantly increased with a P value of 0.0007. For UVC treated *cep-1(gk138)* mutant worms animals fed L4440, the lesions were significantly increased with a P value of 0.05. For UVC treated *cep-1(gk138)* mutant worms animals fed *bec-1* RNAi, the lesions were significantly increased with a P value of 0.003.

It has been shown that Beclin 1 in mammals is involved in genomic instability and that its levels increase after DNA damage [38], [39]. Embryos with *bec-1(ok691)* and *bec-1* RNAi have an increase in TUNEL-positive cells, which could reflect an increase in DNA damage and cell death [38]. After *bec-1* knockdown for 24 hours, lesions in nuclear DNA were measured with and without UVC treatment to determine if *bec-1* knockdown influenced the amount of DNA damage. After UVC treatment a significant increase in lesions were detected in L4440 fed and *bec-1* RNAi fed animals. The partial RNAi knockdown of *bec-1* (in L4 larvae) resulted in an increase in the number of lesions in wild-type animals but the change had a P value of 0.19 (Fig. 8A). The *cep-1(gk138)* mutants had increases in nuclear DNA lesions after UVC and *bec-1* knockdown that were similar to each other (Fig. 8A).

After studying the treated adult worms that only had knockdown of *bec-1* for 24 hours, we went on to study their progeny. These worms were treated with RNAi against *bec-1* throughout development (Fig. 8B). In wild-type worms, RNAi depletion of *bec-1* throughout development caused a significant increase in nuclear DNA lesions (Fig. 8B). In this case the loss of *bec-1* in conjunction with UVC significantly increased the nuclear lesions in the *cep-1(gk138)* loss of function animals (Fig. 8B). In *cep-1(gk138)* mutant animals, the additive effect of the *bec-1* knockdown and UVC exposure significantly increased the nuclear DNA lesions from less than 1 to more than 1.5 lesions per 10 kb with a P value of 0.025 (Fig. 8B). This suggests a cross-talk between CEP-1 and BEC-1 in DNA repair to improve genomic stability.

## Discussion

The loss of functional p53 often occurs in human tumors thus allowing for the survival of cells which lack proper DNA damage checkpoints, cell cycle arrest and cell death [40]. p53 is conserved in its activation of cell death in different lower organisms including *C. elegans* and *Drosophila* [2], [3], [41]. Additionally, p53 in *C. elegans* is involved in UVC mediated germline cell cycle arrest and the regulation of autophagy [24], [42]. The loss of BEC-1 in *C. elegans* causes increased apoptosis and DNA damage [14] and DNA damage increases BEC-1::GFP levels [39]. Herein, is evidence that CEP-1/p53 and BEC-1 in *C. elegans* were involved in maintaining genomic integrity, therefore furthering the conservation of CEP-1/p53 and BEC-1 in *C. elegans*.

p53, in mammals, has been well described as responding to DNA damage to assist in DNA repair and to initiate signal transduction cascades for the activation of cell cycle arrest and cell death [40]. We have reported here, for the first time, that after UVC irradiation and DMC chemotherapeutic treatment, CEP-1/p53 is involved in DNA repair. UVC exposure causes thymine dimers and, if not repaired, can eventually cause DNA double strand breaks. DMC is an alkylating agent which causes DNA adducts and DNA crosslinks, at high frequency and also p53-independent cell death [22], [23]. While *cep-1(gk138)* mutant animals do not have increased cell death after UVC exposure, we observed a significant increase in UVC induced nuclear DNA damage that was not effectively repaired (Fig. 2). The lack of DNA repair provoked a decrease in egg viability in *cep-1(gk138)* animals (Fig. 3). Therefore CEP-1/p53 in *C. elegans*, similar to p53 in mammals, participates in DNA repair.

We previously showed that DMC induces p53-independent cell death and DNA damage in human cancer cells [22], [23]. Very few pharmacological agents effectively enter into *C. elegans,* because of the thick cuticle of the animal, coupled with the animal’s extensive xenobiotic efflux pumps [33]. We scored for germline cell death over a range of DMC concentrations. In *C. elegans*, DMC promoted germline cell death in the *cep-1(gk138)* mutants and not in the wild-type animals (Figs. 3B and 3C). Furthermore, after five hours of treatment, the wild-type animals had less DNA damage from DMC, than the *cep-1(gk138)* mutants but neither population had lesions detected in the significance range detected for UVC (Fig. 3A). This induced death indicated that DMC was taken up by the animals, when administered on plates at high concentrations, for short treatments, and the low level of lesions detected suggested that active DNA repair was ongoing. The increased sensitivity of the *cep-1(gk138)* mutant germline to DMC treatment was probably due to the reduced capability of DNA repair. Moreover, the increase in drug concentration appeared to cause an increase in the xenobiotic efflux pumps as a reduction in cell death was seen when the DMC concentration was increased from 1 mM to 2 mM. Overall *C. elegans* are excellent at evading the toxicity of chemotherapeutic DNA damaging drugs.

Animals with germline tumors were subjected to UVC treatment to examine the influence of CEP-1/p53 activity on developing tumors. We discovered that CEP-1/p53 was transcriptionally functional in the *glp-1(ar202gf)* tumor animals with very little nuclear damage observed after immediate UVC-induced DNA damage (Fig. 4). This was in striking contrast to the increased nuclear DNA damage lesions observed after UVC treatment of the *cep-1(gk138); glp-1(ar202gf)* mutants (Fig. 4A). The germ line tumors are quickly dividing with high levels of DNA replication and this possibly couples with CEP-1 activity in *glp-1(ar202gf)* animals to repair the actively replicating genome directly after UVC-mediated DNA damage. Interestingly, 24 hours after UVC-mediated damage we observed complete lesion removal in both populations (Fig. 4C). UVC treatment of *glp-1(ar202gf)* animals with functional CEP-1/p53, had tumors that decreased in size. However, the UVC treatment of *cep-1(gk138); glp-1(ar202gf)* mutant animals resulted in an increase in tumor size, presumably due to the animals sustaining an increase in genomic instability following lesion removal (Fig. 6). This confirmed the involvement of CEP-1/p53 in nuclear damage repair and the susceptibility of *cep-1(gk138)* mutant tumors to sustain more genomic instability, which resulted in increased tumor sizes. No increase in apoptosis was detected in *glp-1(ar202gf)* as compared to *cep-1(gk138); glp-1(ar202gf)* mutants.

In wild-type *C. elegans*, one-third of all cells are somatic, and the remaining two-thirds, are actively dividing germ cells. Therefore the source of DNA damage, we observed after UVC treatment, was potentially in the somatic cells, as well as in the germ line. The PCR reactions to measure DNA lesions do not distinguish whether the DNA lesions occur in somatic or germ cells, but rather are primer specific, allowing us to compare the amplicons between the nuclear genomes [25]. Using a *glp-1(q224lf)* mutant strain that had no germ cells at 25°C, we observed that CEP-1/p53 was activated in the somatic cells (Fig. 5). Furthermore, when there was no functional CEP-1/p53 present, in *glp-1(q224lf)* loss of function mutant animals, they had reduced DNA repair (Fig. 5). Our data did not support the previously published work, using a different germline null animal and exposure to ionizing radiation (IR) to induce CEP-1 downstream target gene activation [7]. Previously, it was shown that *glp-1(q224lf)* mutant animals have nuclear DNA repair after UVC at slower rates than wild-type [21] and germline null animals have no *egl-1* activation after IR [7]. The difference in our results could be due to the differences in lesion types and the type of CEP-1 activation. Our data indicate a previously unreported role for CEP-1 in DNA repair of UVC lesions in the soma, as well as in the germ line.

BEC-1 in *C. elegans* has important roles in autophagy, cell death and DNA damage, but its relationship with p53 has yet to be clearly defined. We observed that adult worms with *bec-1* knockdown, for a short period of time, had increased cell death in the germ line that was CEP-1-dependent, and the depletion of *bec-1* only slightly increased *egl-1* mRNA induction (Fig. 7). While we observed CEP-1-dependent cell death, caused by *bec-1* knockdown in adult animals, we did not observe increased DNA damage lesions in these animals (Fig. 8A). Therefore, there may be cross-talk between CEP-1 and BEC-1 activities that regulates CEP-1 DNA repair functions. In mammals, Beclin 1 associates with the phosphatidylinositol 3-Kinase/Vps34 to activate the formation of the pre-autophagosomal membrane [43]. The reduction of BEC-1 activity in the RNAi depleted animals might function to activate a CEP-1 dependent death pathway in a way that has not yet been elucidated. In a Myc driven tumor model, the inhibition of autophagy activates p53-mediated cell death [44]. This is reminiscent of what we observed in *C. elegans*. On the other hand, RNAi depletion of *bec-1* in the progeny increased nuclear DNA-damage lesions in UVC treated, animals with a more profound outcome observed in *cep-1(gk138)* mutant animals (Fig. 8B). This suggests that in the absence of CEP-1 and BEC-1 there is less DNA repair and less DNA repair clearance.

Here, we have demonstrated novel roles for CEP-1 and BEC-1 in cell death, and the removal of DNA damage. The increased sensitivity of the *cep-1(gk138)* mutant germline to DNA damage, following UVC treatment, or DMC chemotherapy treatment, resulted in decreased embryonic viability. This strongly suggests that CEP-1 participates in improving the germ line genomic integrity to increase fecundity. This is not surprising as p53, p63, and p73 have all been shown to be guardians of female reproduction in mammals [45], [46]. Autophagy mitigates DNA damage in mammary tumorigenesis and *beclin 1* controls this process [38]. Our data suggest a cooperative role for BEC-1 in DNA damage repair and apoptotic corpse clearance. An increase in the number of cell corpses has been reported to occur in animals depleted of *bec-1* activity throughout development [15], but the dependency of cell death on CEP-1 after a partial knockdown of *bec-1* resulting from the RNAi depletion of adults (starting at the L4 stage), is a new finding. While the exact role of BEC-1 on CEP-1, and vice versa, is unknown we observed that both participated in DNA repair, cell death, and cross-talk in the *C. elegans* germline.

## Materials and Methods

### Growth Media and Strains

All strains were maintained and constructed using standard methods and were grown on nematode growth media (NGM) at either 15°, 20° or 25°C [47]. One liter of NGM includes: 3 g NaCl (Fisher), 17 g agar (Fisher), 2.5 g bactopeptone (Becton, Dickinson and Company), 1 mL cholesterol (Sigma, 5 mg/mL in 95% Ethanol), 1 mL 1 M CaCl2, 1 mL 1 M MgSO4, 25 mL 1 M potassium phosphate pH 6. The plates were dried and the E. coli strain OP50 was spread for worms to eat. Seeded plates were left overnight at room temperature and could be used for three weeks. M9 buffer was prepared using 3 g KH2PO4, 6 g Na2HPO4, 5 g NaCl, 1 ml 1 M MgSO4, H2O to 1 L. Sterilize by autoclaving. The wild-type strain used was *C. elegans* variety Bristol (N2). The following mutant strains were used as well: *bcIs39[P(lim-7)ced-1::GFP+lin-15(+)]* (expression of functional CED-1::GFP fusion protein in the sheath cells, MD701 [48], *cep-1(gk138)* (1660 bp deletion, TJ1), *glp-1(q224lf)* (base substitution G1043E, JK1107), and *glp-1(ar202gf)* (base substitution G529E, GC833). Strains were provided by the *Caenorhabditis Genetics Center*. *cep-1(gk138); bcIs39[P(lim-7)ced-1::GFP+lin-15(+)]* (JBC1), *cep-1(gk138)* and *glp-1(ar202gf)* (JBC2) were constructed by the Bargonetti Laboratory according to methods described [49]. The *glp-1(q224lf), glp-1(ar202gf)* and *cep-1(gk138); glp-1(ar202gf)* were maintained at 15°C and moved to 25°C as eggs to promote a germline-tumor or germline null phenotype.

### RNAi Feeding

RNAi plates were prepared using the normal NGM method with a final concentration of 2 mM (*bec-1*) or 6 mM (*cep-1*) of IPTG (Fermentas) and 50 µg/mL Carbenicillin (Sigma). For *cep-1* knockdown, L4 worms were placed on RNAi seeded plates with bacteria containing the L4440 control plasmid or *cep-1* RNAi plasmid a generation before treatment (generous gifts from the Gartner laboratory). This bacterium was validated for the ability to inhibit basal, and DNA damage induced, *egl-1* and *ced-13* (data not shown). For partial *bec-1* (treated adult) knockdown L4 larvae were placed on RNAi seeded plates with bacteria containing the L4440 control plasmid or *bec-1* RNAi plasmid (from the Ahringer library, generous gifts from the Melendez laboratory), and were analyzed 24 or 48 hours post L4 as indicated. For animals *bec-1* RNAi depleted throughout development, adults were placed on RNAi plates and their progeny grown on RNAi plates and analyzed 24 or 48 hours post L4 as indicated.

### UVC Treatments

UVC treatments were done with a SpectroLinker. Worms were rinsed off plates with M9 and added to plates without bacteria. Worms were then exposed to 50 or 100 J/m^2^ of UVC or left untreated as negative controls. For RNA extraction, the worms were rinsed off and placed back onto seeded NGM plates and allowed to recover for four hours, after which the worms were washed, collected, and frozen at −80°C. For germline cell death analysis, worms were placed back onto NGM plates for 24 hours and then scored. For DNA lesion analysis, the worms were placed in lysis buffer directly after treatment. For DNA damage repair analysis, worms were frozen four, eight, sixteen or twenty-four hours after treatment. All worms were frozen at −80°C until processing.

### RNA Isolation from *C. elegans* and Quantitative Reverse Transcription PCR (Q-RTPCR)

Worms were centrifuged at 1,500 rpm for 4 minutes at 4°C, washed twice with M9, and the RNA was isolated using QIAshredder columns and the RNeasy Mini Kit (Qiagen) following the manufacturer’s protocol. 60 µL of beta mercaptoethanol (BME, Sigma) was added to 6 mL of RNeasy Lysis Buffer (RLT) and 600 µL of this solution, was added to the worm pellets. Worms were incubated for 10 minutes and then spun for 2 minutes at 13,000 RPM in the QIAshredder. 600 µL of 70% ethanol was added to the flow through and spun on a collecting column. The collecting column was washed with 350 µL of RNeasy Wash Buffer (RW1) and then incubated with 10 µL DNase and 70 µL of RNeasy DNA Digest Buffer (RDD) for 15 minutes. The collecting column was washed again with 350 µL of RW1 and then 500 µL of RNeasy Clean Up Buffer (RPE), each spun for 30 seconds. A last spin of 500 µL of RPE was done for 2 minutes and the membrane was dried. The collecting column was placed on a fresh 1.5 mL eppendorf collecting tube and the RNA was eluted with 30 µL of RNase free water, and spun at 10,000 RPM for 1 minute. The RNA was only used if the yield was greater than 100 ng/µL. RNA was stored at −80°C. 300 ng of RNA in 25 µL of volume was used for cDNA synthesis using the High Capacity cDNA Archive Kit reagents as described by the manufacturer (Applied Biosystems). 25 µL of the reverse transcription (RT) master mix contained 1x RT buffer, 1x dNTP’s, 1x random primers and 1 U/µL of Multiscribe Reverse Transcriptase. The reaction was incubated at 25°C for 10 minutes and then at 37°C for 2.5 hours. The cDNA was stored at 4°C for short term and −20°C for long term. Amplification of gene transcripts by quantitative PCR with 0.125 µL of primers for *egl-1*, *ced-13* and *tbg-1* (Operon and Applied Biosystems, see sequences below, final primer concentration of 0.05 pmol/µL) [8] combined with the 3 µL of cDNA and 12.5 µL of SYBR Green PCR Master Mix (volume brought up to 25 µL with nuclease free water) (Applied Biosystems) was carried out following the program: one cycle, 2 minutes, 50°C; one cycle, 10 minutes, 94°C; and 40 cycles or 15 seconds at 94°C and 1 minute at 60°C for *egl-1* and *tbg-1* or 54.6°C for *ced-13* in a 7500 Sequence Detection System (Operon and Applied Biosystems). Primers for *tbg-1* were forward: 5′-cgtcatcagcctggtagaaca-3′ and reverse: 5′-tgatgactgtccacgttgga-3′. Primers for *egl-1* were forward: 5′-tactcctcgtctcaggactt-3′ and reverse: 5′-catcgaagtcatcgcacat-3′. Primers for *ced-13* were forward: 5′-acggtgtttgagttgcaagc-3′ and reverse: 5′-gtcgtacaagcgtgatggat-3′.

### Induction of Germ Cell Apoptosis using DMC

10-decarbamyl-mitomycin C (DMC) was a gift from Dr. Maria Tomasz. 2 mM was diluted to desired concentration in 30% methanol. Except for the concentration curve, all DMC experiments were carried out using 1 mM DMC. 45 µL DMC or 45 µL 30% Methanol were placed onto the bacteria of seeded NGM plates. After three hours, young adult worms were moved to treated plates and left either over-night or for five hours. Germline cell death was analyzed at the end of treatments and worms were frozen in lysis buffer for analysis using the lesion assay.

### Germline Apoptosis Scoring

Analyses of germline apoptosis were done using the reporter CED-1::GFP for engulfed apoptotic nuclei [50], by mounting treated worms after a 24 hour recovery after UVC or immediately after DMC feeding. Slide pads were prepared with a drop of 2% agarose flattened by another slide, and 5 µL of 3 mM levamisole diluted in M9 Buffer was added to anesthetize worms. Normarski and green fluorescent images were taken using an ApoTome Zeiss microscope taking pictures of each plane of the germ line (1 µm thick with an average of 25 slices per worm). Images were saved using a generic label and later scored blindly by two independent scientists. Each scientist counted CED-1::GFP positive cells from the images, and although the exact counts of CED-1::GFP positive cells did not always match, the average trend in the number of CED-1::GFP positive cells, per treatment, were always consistent for the two scientists.

### DNA Lesion Assay

Five worms were placed in 150 µL of lysis buffer (1x rTth XL DNA polymerase buffer and 1 mg/mL proteinase K (Applied Biosystems and Sigma) after treatment and frozen for at least 10 minutes at −80°C. The lysis step included 1 hour at 5°C, with a vortex after 5 minutes, and 15 minutes at 95°C. The 50 µL QPCR (quantitative polymerase chain reactions) mixtures contained the following: 9.6 µL sterile de-ionized water, 15 µL 3.3× rTth XL DNA polymerase buffer, 5 µL of 1 mg/mL bovine serum albumin, 4 µL dNTPs (2.5 mmol/L of each), 2.4 µL 25 mM MgO(Ac)2, 2 µL of each primer, and 5 µL of 2 ng/µL genomic DNA template. All primers were used at 10 µmol/L. rTth XL DNA polymerase was diluted in 1× buffer, and 5 µL was added in a hot start procedure. The 3.3× buffer, MgO(Ac)2, and rTth XL DNA polymerase were from the GeneAmp XL PCR kit (Applied Biosystems). The cycling conditions for the small nuclear target was as follows: 1 cycle of 75°C for 2 minutes; 1 cycle of 94°C for 1 minutes; 29 cycles of 94°C for 15 seconds, 63°C for 45 seconds, and 72°C for 30 seconds, and 1 cycle of 72°C for 5 minutes. The cycling conditions for the large nuclear targets was: 1 cycle of 75°C for 2 minutes; 1 cycle of 94°C for 1 minutes; 31 cycles of 94°C for 15 seconds and 68°C for 12 minutes, and 1 cycle of 72°C for 10 minutes [21]. The primers used were as following (IDT): **1)** Small Nuclear (polymerase ε target): forward 5′ tcc cgt cta ttg cag gtc ttt cca 3′ and reverse 5′ gac gcg cac gat atc tcg att ttc3′; and **2)** Large Nuclear (*unc-2*): forward 5′ tgg ctg gaa cga acc gaa cca t 3′ and reverse 5′ ggc ggt tgt gga gtg tgg gaa g 3′. The DNA quantity was measured after QPCR using PicoGreen dye (Invitrogen). Each sample was measured in duplicate. 90 µL of 1xTE buffer, 10 µL of DNA and 100 µL of a solution of PicoGreen reagent (5 µL of reagent per milliliter of 1x TE) were added to each well. The sample was mixed and incubated at room temperature for 10 minutes in the dark. The fluorescence was read using 485 nm of excitation and 530 nm for emission using a Spectra Max Gemini EM detector (Molecular probes). Values were normalized to untreated samples using a Poisson distribution [51]. Blanks were subtracted from all values. Small nuclear numbers where divided by the average of the small values in their experiment, termed “factor”. The large nuclear values were divided by the factor. The result was divided by the untreated value, and yielded a relative amplification value. The –LN of the relative amplification value was then calculated and this value was multiplied by 10 and divided by 10.939 to determine the lesions/10 kb value.

### Tumor Growth

Synchronized adult worms were placed on plates and allowed to lay eggs. After about 100 eggs were laid the adults were picked off the plate, and the egg-lay sync plates were placed at 25°C. Each day until the worms reach the L4 stage, the egg-lay synchronized plates were looked through, and any worms that were obviously younger or older than the majority of the worms were picked off the plates. Three days after the egg-lay sync (when the worms have reached the L4 stage), the worms were moved to an unseeded plate and each strain was treated with 100 J/m^2^ of UVC, and placed back at 25°C. The worms were allowed to recover for four days after UVC treatment. Four days after UVC treatment, the worms were washed off the plates, fixed with ethanol, and stained with DAPI (Vectashield). Under the microscope, pictures were taken of any intact worms (worms that have not exploded due to the size of their tumor and/or the fixing and staining process), and the gonad tumor progression was determined. The gonad tumor progression was determined by measuring the distance between the anterior-most point of the tumor, and the anterior-most point of the mouth. Cell death was analyzed four hours after UVC treatment and corpses were identified by SYTO-12 staining.

### Statistics

The Graph Pad Prism software version 6.0 d was used to analyze all data. The significance was computed by unpaired, two-tailed student t test. The P values are presented in the figure legends.
